# Supplementary material for: A New Experimental Design to Examine Cognitive Biases for Gastrointestinal Related Stimuli in Children and Adolescents
Source: Children (Basel). 2023 Aug 1;10(8):1327. doi: 10.3390/children10081327 (PMC10453649; doi:10.3390/children10081327)
Supplement: Supplementary file 1 [file children-10-01327-s001.zip › children-2437955-supplementary.pdf]

Supplementary Material

Table S1. Symptom words used in encoding and recognition phases.

Encoding phase	Foils for recognition
**General symptoms**	
Hovedpine<br>(CSSI: Headaches)	Trykken for brystet<br>(HNST: Chest pain)
Svimmelhed<br>(CSSI: Faintness or dizziness)	Smerter i knæ, albuer og andre led<br>(CSSI: Pain in your knees, elbows or other joints)
Træthed<br>(Tiredness (CSI: Low energy/slowed down))	Feber<br>(HNST: Fever)
Ømme muskler<br>(CSSI: Sore muscles)	Følelsesløshed eller prikken i dele af kroppen<br>(CSSI: Numbness or tingling in parts of your body)
Hoste<br>(HNST: Coughing)	Sløret syn<br>(CSSI: Blurred vision)
Svært ved at trække vejret<br>(CSSI: Trouble getting your breath)	Svaghed<br>(CSSI: Weakness in parts of your body)
Svedeture eller kulderystelser<br>(CSSI: Hot or cold spells)	Udmattet<br>(Exhausted)
Smerter i ryggen<br>(CSSI: Pains in your lower back)	Nyse<br>(HNST: Sneezing)
Smerter i arme og ben<br>(CSSI: Pain in your arms or legs)	Ondt i ørerne<br>(HNST: Earache)
Hjertebanken<br>(CSSI: Your heart beating too fast)	Smerte i hjerte eller bryst<br>(CSSI: Pain in your heart or chest)
**Encoding phase**	**Foils for recognition**
**Gastrointestinal symptoms**	
Mavesmerter<br>(PedsQL (GSS): Pain in abdomen/stomach)	Trykken i maven<br>(Stomach pressure)
Diarre<br>(PedsQLGSS: Diarrhea)	Ikke kunne tåle mad<br>(CSSI: Food makes you sick)
Forstoppelse<br>(PedsQL (GSS): Constipation)	Klump i halsen<br>(Lump in the throat)
Kvalme<br>(PedsQL (GSS): Nausea)	Bøvsen<br>(Burping)
Opkast<br>(PedsQL (GSS): Vomiting)	Hård mave<br>(Difficulty passing stools)
Ubehag i maven<br>(PedsQL (GSS): Discomfort in stomach/abdomen)	Mavekramper<br>(Stomach cramps)
Besvær med at synke<br>(CSSI: Difficulty swallowing)	Sure opstød<br>(Heart burn/pyrosis)
Oppustethed<br>(PedsQL (GSS): Bloating)	Rumlen i maven<br>(Stomach rumbling)
Dårlig appetit<br>(PedsQL (GSS): Not feeling hungry)	Prutte<br>(Farting/flatulence)
Luft i maven<br>(Trapped gas)	Tynd mave<br>(Loose and watery stools)

Table S1: Overview of Danish symptom words used in encoding and recognition phase with corresponding English words in parenthesis. CSSI: Children's Somatic Symptoms Inventory. HNST: Health Norms Sorting Task. PedsQL (GSS): Pediatric Quality of Life, Gastrointestinal Symptoms Scale.

Table S2. Description of pictures from encoding and recognition phases.

Encoding phase	Foils for recognition
**Food**	
Picture 19: Boy lying on a couch, empty cups and food wrappers on the floor	Picture 21
Picture 93: People ordering food at a fast food counter	Picture 81
Picture 77: Girl eating her breakfast)	Picture 55
Picture 178: Girl handing an ice cream to a boy	Picture 136
Picture 153: Two adults and two children having a meal together	Picture 44
**School**	
Picture 143: Woman and girls using computers	Picture 7
Picture 151: Man opening the door to two children	Picture 152
Picture 30: Girl sitting alone, while two other girls are chatting behind her	Picture 23
Picture 149: Two girls and a boy writing in books	Picture 45
Picture 63: People in a library	Picture 48
**Fun**	
Picture 139: Girl playing a game while a boy looks on	Picture 52
Picture 197: Two boys playing with water guns	Picture 202
Picture 191: Children playing with skipping rope	Picture 117
Picture 190: Children on a bike and a skateboard	Picture 99
Picture 194: People at a birthday party	Picture 177

Table S2: Overview of pictures from encoding and recognition phases. Numbers and descriptions according to the PiSCES Database (Teh, E. J., Yap, M. J., & Liow, S. J. R. (2018). PiSCES: Pictures with social context and emotional scenes with norms for emotional valence, intensity, and social engagement. Behav Res Methods, 50*(5), 1793-1805. https://doi.org/10.3758/s13428-017-0947-x)*
